# Supplementary material for: Assessing movement quality in individuals with Duchenne muscular dystrophy utilizing accelerometry: Comparisons with healthy controls
Source: PLOS Digit Health. 2026 Jun 2;5(6):e0001315. doi: 10.1371/journal.pdig.0001315 (PMC13229295; doi:10.1371/journal.pdig.0001315)
Supplement: Attachment — Submitted filename: Response_to_Reviewers_28Apr2026.docx [file pdig.0001315.s002.docx]

**28 April 2026**

Reviewer #1

1. In the Abstract, it is recommended to provide sufficient information about the research methodology, including which descriptive data were recorded for each group, which accelerometer was used, the data collection process, etc.

Thank you for this recommendation. The methods (Lines 55-61) portion of the abstract has been revised to add information on the specific device models utilized in each cohort and the descriptive and inferential statistics analyzed in both the DMD and Healthy Control cohorts.

2. In the Abstract, it is recommended to present the research results with numerical data, including the descriptors of the groups and comparison results with healthy individuals.

We appreciate this feedback. A p-value reference was added to Line 63 indicating the statistical significance of the median accelerometer measure differences. Additionally, the median and IQR data for all cohorts were added to lines 65-66 for the specific focus on the increased median movement frequency in DMD relative to healthy controls. Lastly, correlation coefficients and P-values were added to lines 67-68 for the analysis between counts per minute and jerk and mean frequency.

3. In the Method section, the distribution in Table 3 could be corrected by providing separate data for all ethnic groups.

We have rearranged Table 2 as requested to more clearly separate ethnicity data in both DMD and healthy controls.

4. In the Methods section, was ambulatory status recorded only as walker/non-walker? Or was it assessed according to any other classification? If applicable, a comparison according to ambulatory level could also be added, so that information on which parameters of the quantity and quality of movement changed at different ambulatory levels would provide valuable information to rehabilitation specialists.

This is an important question. Unfortunately ambulatory status for this study was recorded only as walker/non-walker, so we do not have a further classification available to compare by. We have added a sentence clarifying the classification of ambulatory status utilized in this study to Lines 157-158 and acknowledged the limitation of this classification in revised lines 405-408.

Reviewer #2

1. It would be interesting to specify the participants' Signs, since in ambulatory individuals, the Signs can range from 1 to 6, and in non-ambulatory individuals from 7 to 10. This is a point to be included in the characterization of the sample and to verify possible relationships, since this scale indicates the staging of the disease - the level of functionality of the individual with DMD.

Unfortunately (see response to Reviewer 1 #4 above) due to the limited classification data of participant ambulatory status available in this cohort, we are unable to specify the participants’ Signs, but we agree this further stratification could certainly be of future clinical utility. We have revised lines 405-408 of the limitations to acknowledge the challenge of our present ambulatory classification method.

2. Regarding the results, I suggest reviewing the formatting of all tables, as they are presented as charts. Tables have open sides.

Thank you for this feedback. The tables have been reformatted as suggested to contain open sides.

3. Regarding tables 3 and 5, include the mean and standard deviation of the evaluated variables on the same line, for example: mean acceleration and standard deviation of acceleration.

Thank you for raising this point. In the case of our present analysis, the median acceleration, standard deviation of acceleration, and peak acceleration, as well as the mean frequency and standard deviation of frequency, were each analyzed as individual accelerometer measures, as we defined in Table 1. We have represented this respectively in both Table 3 where each measure’s median and IQR was calculated for each cohort, as well as Table 5 where the results of each measure’s univariate regression was shown.

4. In the first part of table 2 and in table 3, indicate what the values in parentheses mean. I believe they are the minimum and maximum values of the studied variables.

Thank you for your feedback on this. The values in parentheses of the first part of table 2 and table 3 represent either the IQR for the studied variables where a median value is presented, or a percentage when a numerical count of participants is presented (such as number of ambulatory participants). Additional statements have been added to the footnotes of Tables 2 and 3 clarifying this.

5. I suggest adding the clinical applicability of the study findings to the discussion. This needs to be explored

Thank you for this suggestion. We have expanded and revised the discussion to highlight the potential clinical utility of accelerometry, particularly its use as a potential outcome measure for clinical trials, as well as its comparisons to the surrogate outcome of skeletal muscle MRI.

6. The discussion will present, as a limitation, the importance of assessing upper limb function in ambulatory and non-ambulatory individuals using clinical scales that indicate upper limb performance and compensations, as well as the compensatory strategies used by this population to maintain function, and how this could be explored in future studies.

We agree that the limited upper limb performance and compensation data in this analysis needs further exploration. Lines 418-426 of the limitation section have been modified to include discussion of the need for expanded analysis of upper limb performance data, such as data from PUL 2.0, and how it could be of particular utility in developing a corrective factor for assistive mobility technologies.

Sincerely,

**Nicholas Joy, B.S.**
